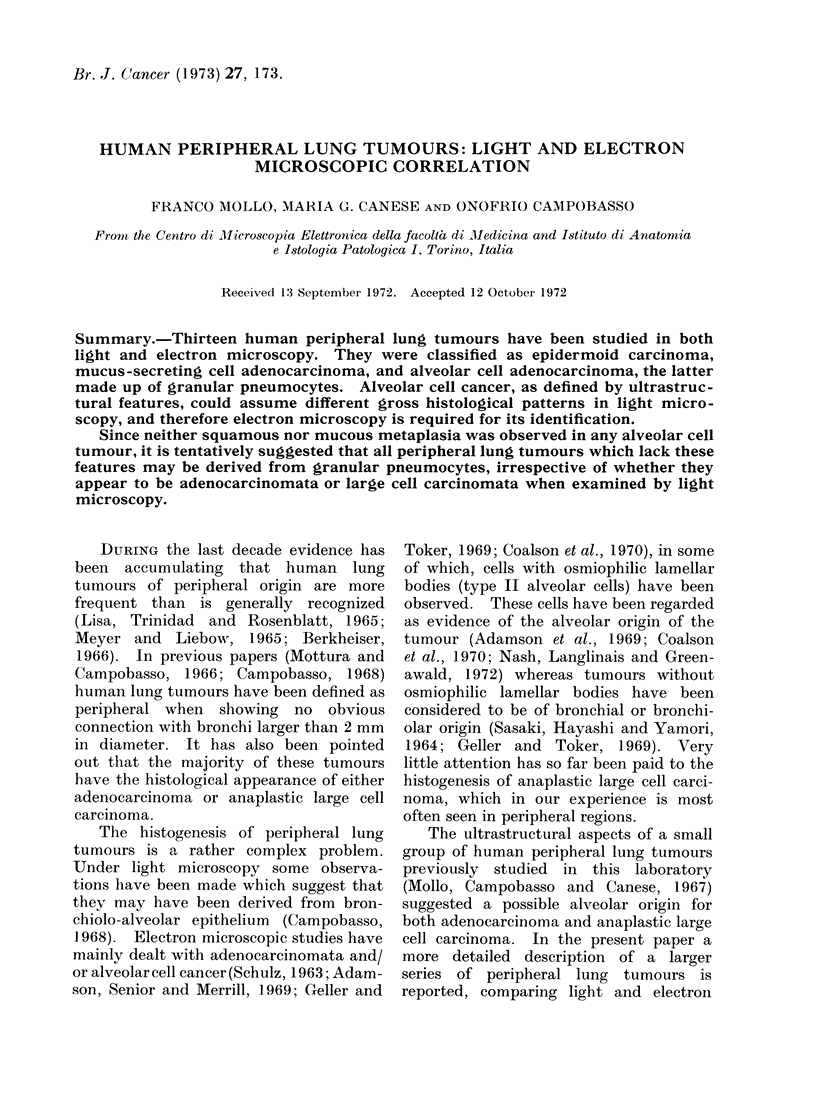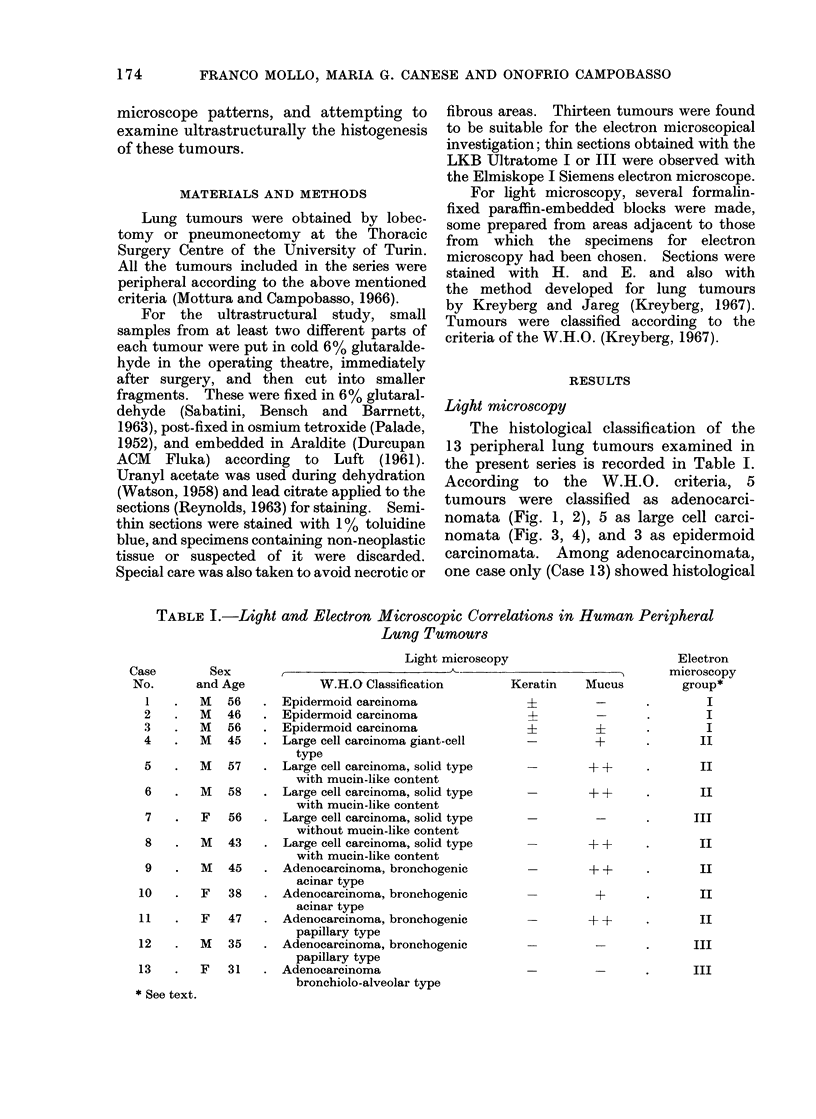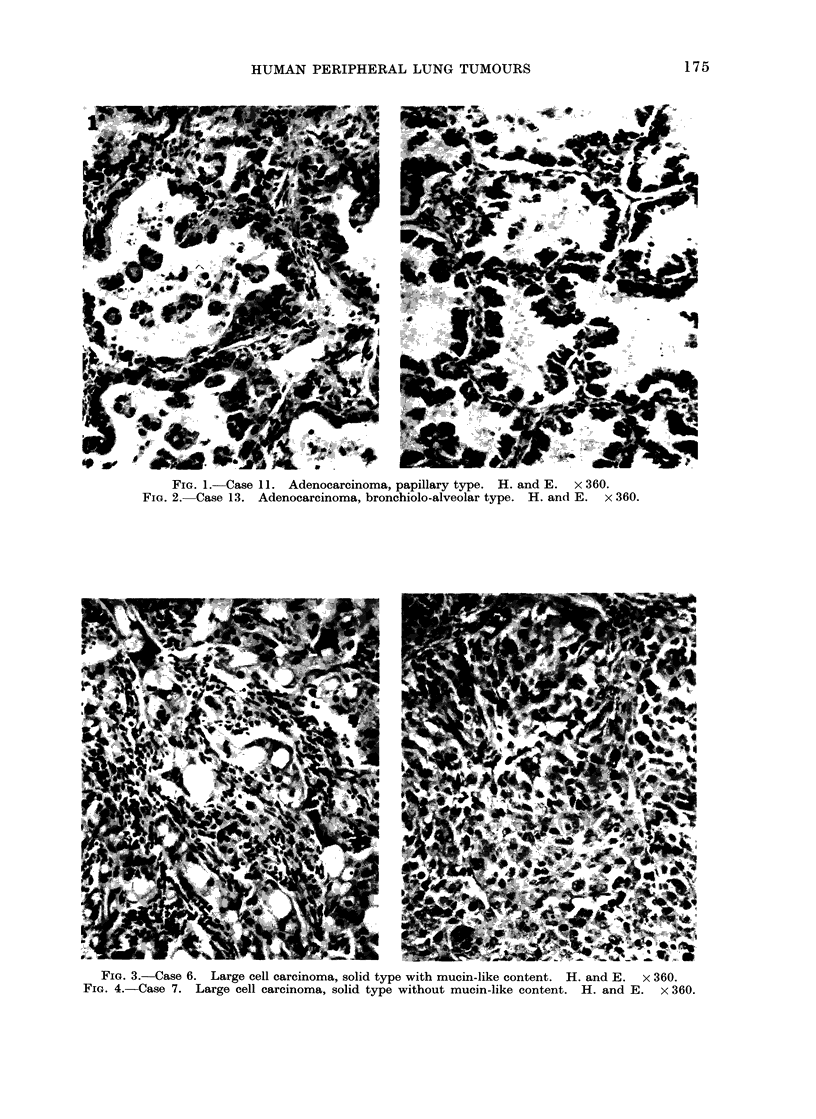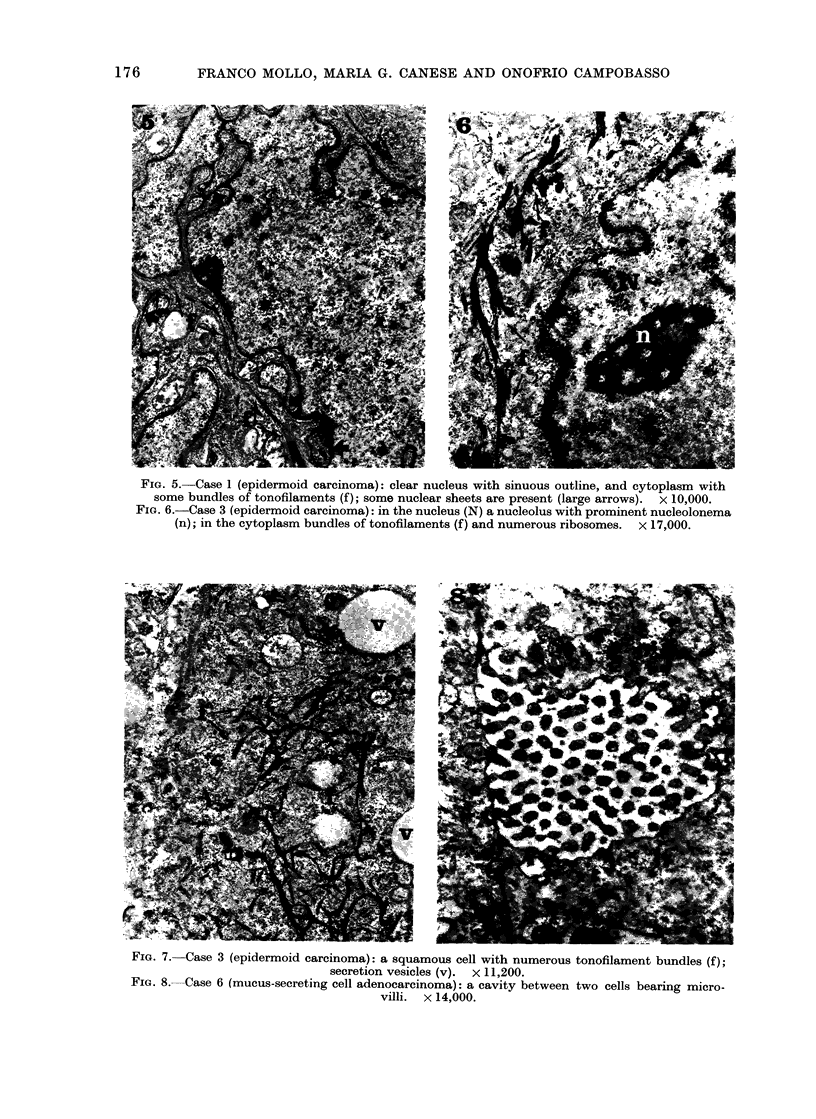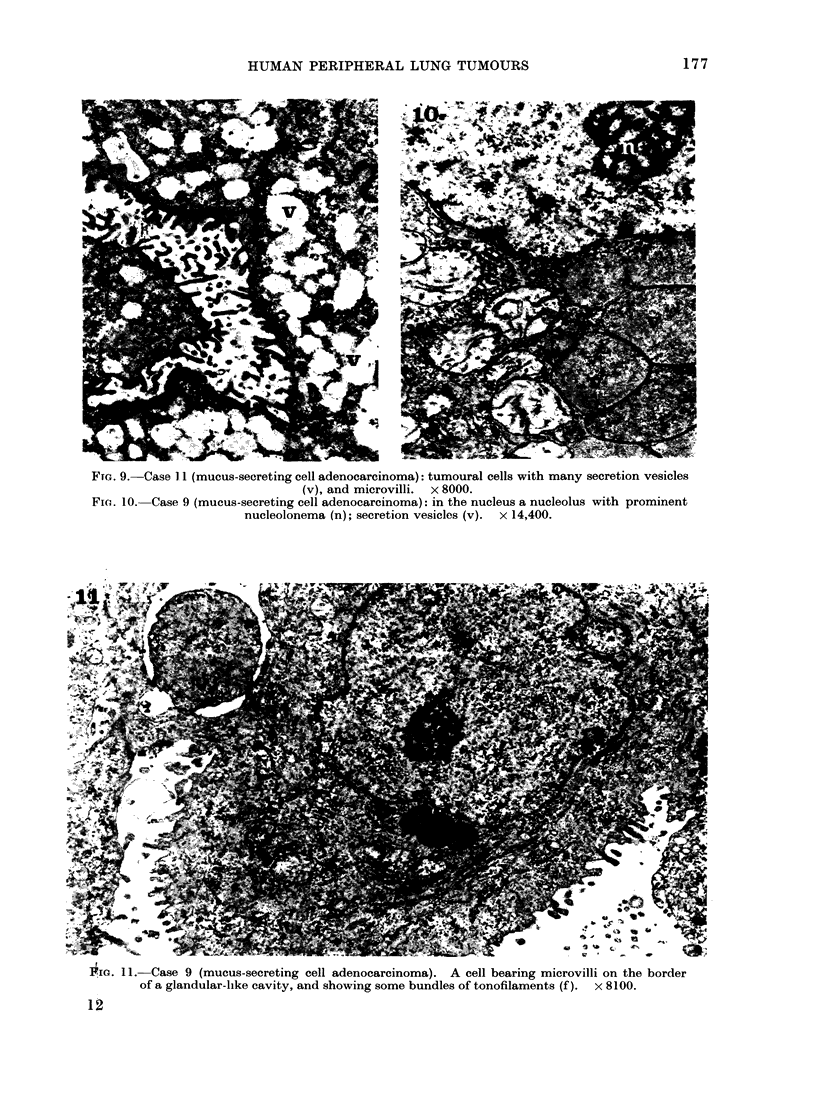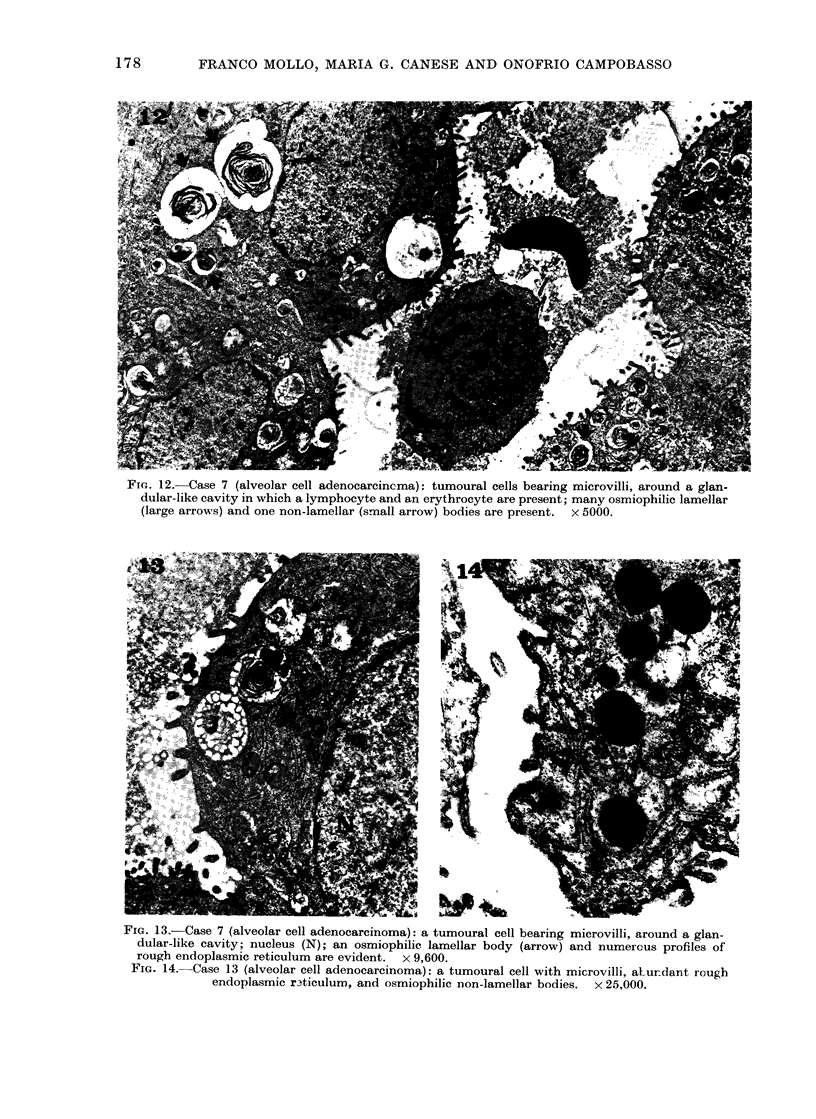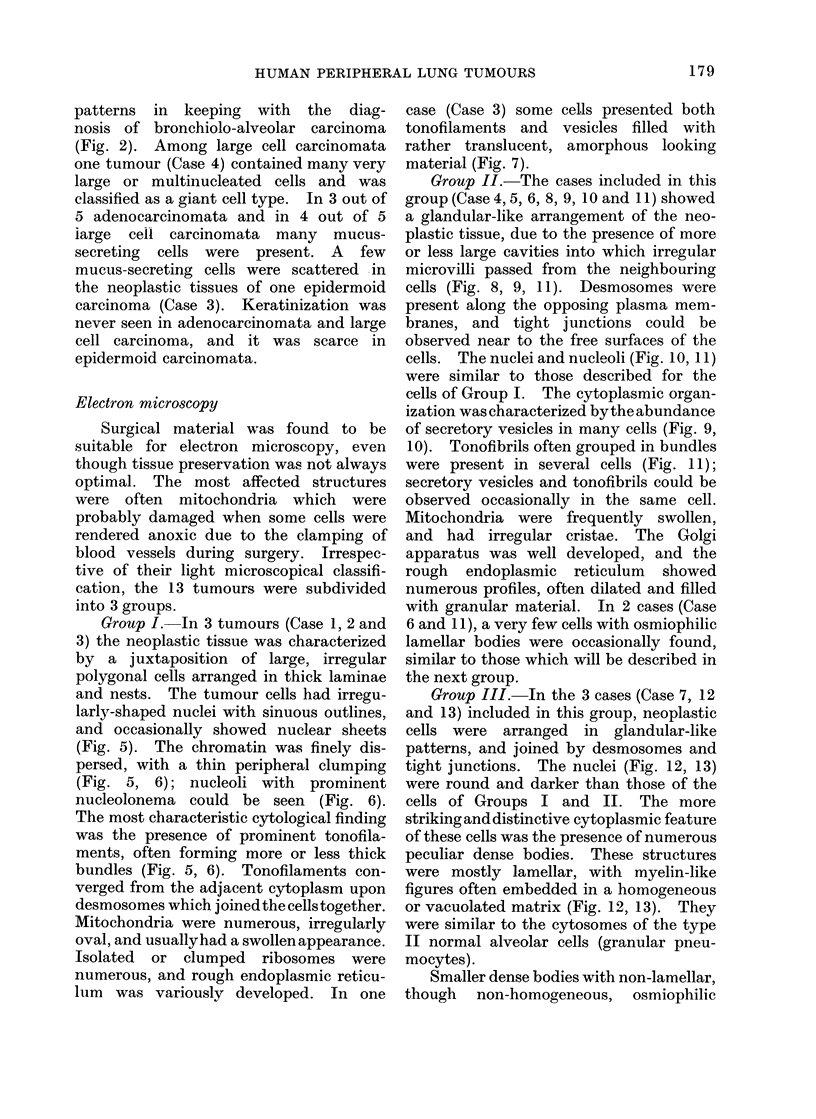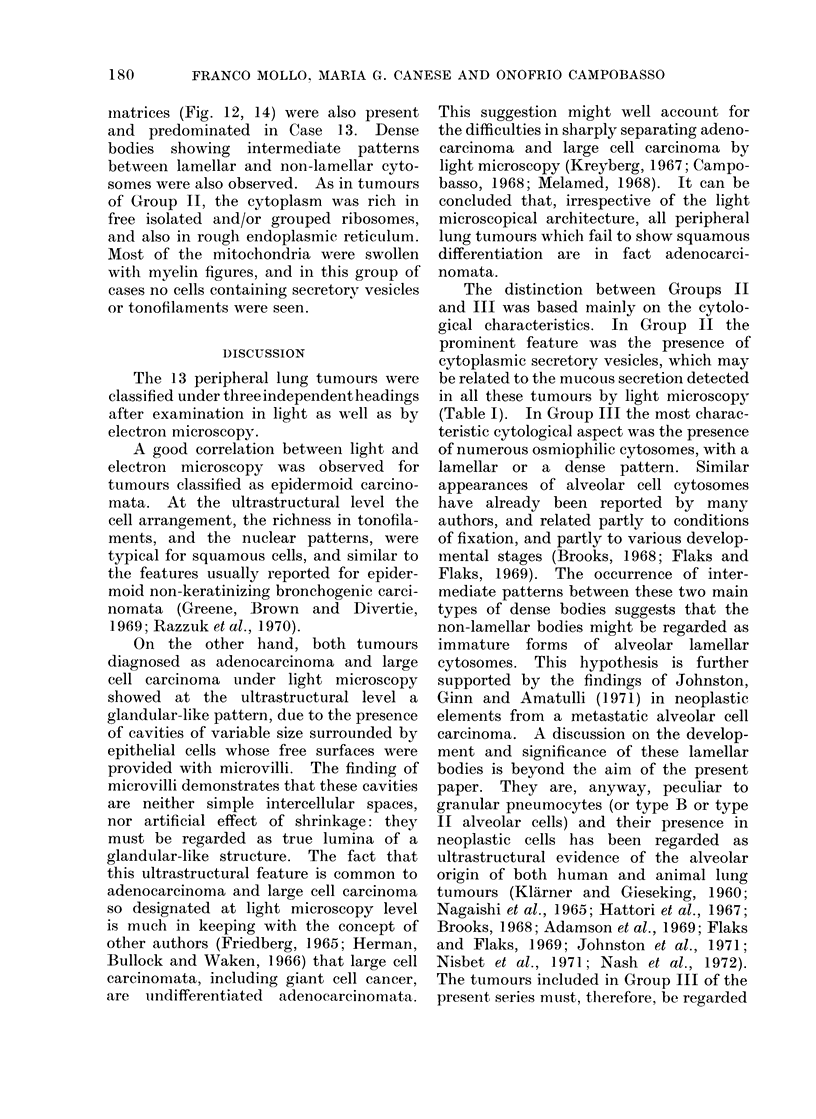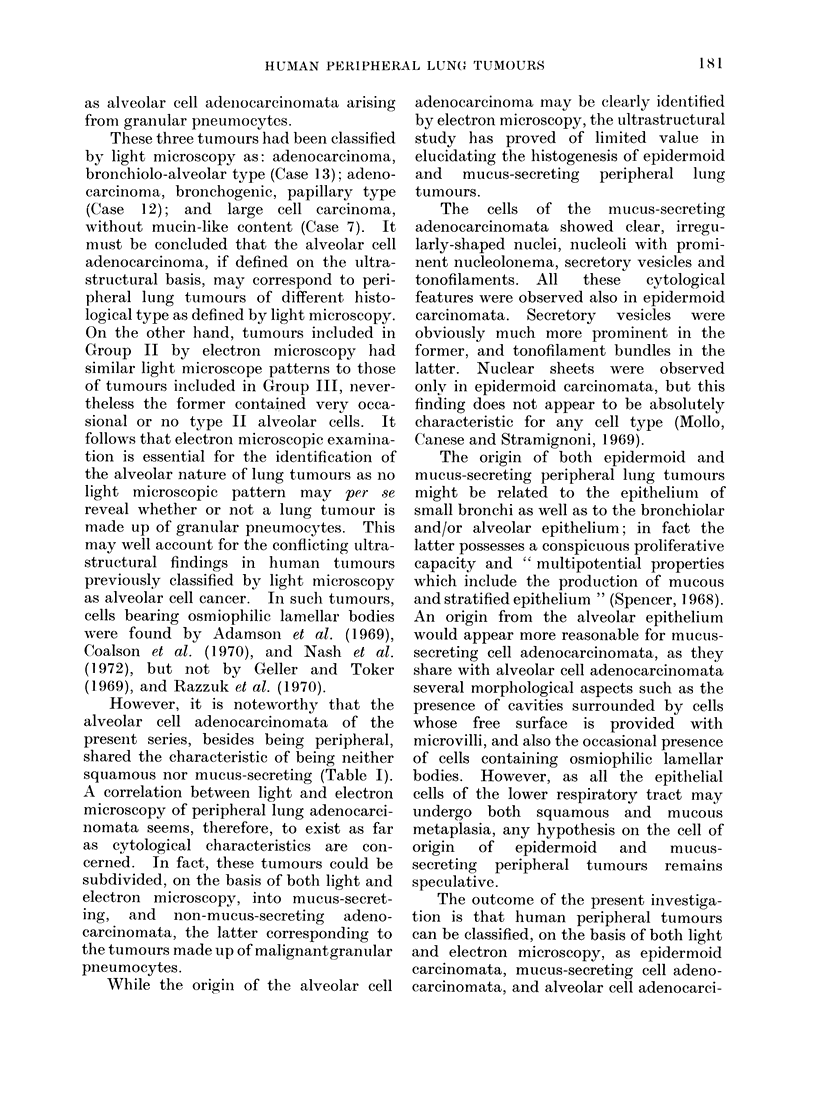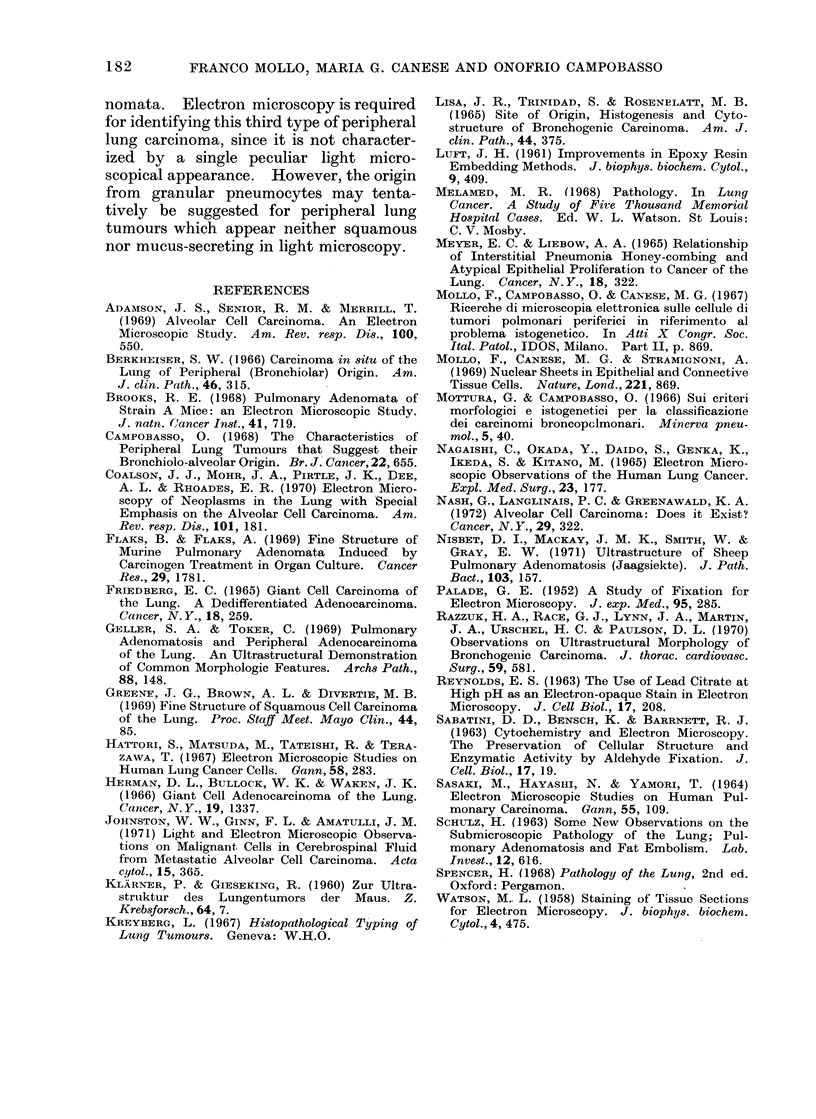# Human Peripheral Lung Tumours: Light and Electron Microscopic Correlation

**DOI:** 10.1038/bjc.1973.21

**Published:** 1973-02

**Authors:** Franco Mollo, Maria G. Canese, Onofrio Campobasso

## Abstract

**Images:**


*Br. J. Cancer* (1973) **27**, 173.

### HUMAN PERIPHERAL LUNG TUMOURS: LIGHT AND ELECTRON MICROSCOPIC CORRELATION

FRANCO MOLLO, MARIA G. CANESE AND ONOFRIO CAMPOBASSO

*From the Centro di Microscopia Elettronica della facoltà di Medicina and Istituto di Anatomia e Istologia Patologica I, Torino, Italia*

Received 13 September 1972. Accepted 12 October 1972

**Summary.—Thirteen human peripheral lung tumours have been studied in both light and electron microscopy. They were classified as epidermoid carcinoma, mucus-secreting cell adenocarcinoma, and alveolar cell adenocarcinoma, the latter made up of granular pneumocytes. Alveolar cell cancer, as defined by ultrastructural features, could assume different gross histological patterns in light microscopy, and therefore electron microscopy is required for its identification.**

**Since neither squamous nor mucous metaplasia was observed in any alveolar cell tumour, it is tentatively suggested that all peripheral lung tumours which lack these features may be derived from granular pneumocytes, irrespective of whether they appear to be adenocarcinomata or large cell carcinomata when examined by light microscopy.**

DURING the last decade evidence has been accumulating that human lung tumours of peripheral origin are more frequent than is generally recognized (Lisa, Trinidad and Rosenblatt, 1965; Meyer and Liebow, 1965; Berkheiser, 1966). In previous papers (Mottura and Campobasso, 1966; Campobasso, 1968) human lung tumours have been defined as peripheral when showing no obvious connection with bronchi larger than 2 mm in diameter. It has also been pointed out that the majority of these tumours have the histological appearance of either adenocarcinoma or anaplastic large cell carcinoma.

The histogenesis of peripheral lung tumours is a rather complex problem. Under light microscopy some observations have been made which suggest that they may have been derived from bronchiolo-alveolar epithelium (Campobasso, 1968). Electron microscopic studies have mainly dealt with adenocarcinomata and/or alveolar cell cancer (Schulz, 1963; Adamson, Senior and Merrill, 1969; Geller and Toker, 1969; Coalson *et al.*, 1970), in some of which, cells with osmiophilic lamellar bodies (type II alveolar cells) have been observed. These cells have been regarded as evidence of the alveolar origin of the tumour (Adamson *et al.*, 1969; Coalson *et al.*, 1970; Nash, Langlinais and Greenawald, 1972) whereas tumours without osmiophilic lamellar bodies have been considered to be of bronchial or bronchiolar origin (Sasaki, Hayashi and Yamori, 1964; Geller and Toker, 1969). Very little attention has so far been paid to the histogenesis of anaplastic large cell carcinoma, which in our experience is most often seen in peripheral regions.

The ultrastructural aspects of a small group of human peripheral lung tumours previously studied in this laboratory (Mollo, Campobasso and Canese, 1967) suggested a possible alveolar origin for both adenocarcinoma and anaplastic large cell carcinoma. In the present paper a more detailed description of a larger series of peripheral lung tumours is reported, comparing light and electron microscope patterns, and attempting to examine ultrastructurally the histogenesis of these tumours.

### MATERIALS AND METHODS

Lung tumours were obtained by lobectomy or pneumonectomy at the Thoracic Surgery Centre of the University of Turin. All the tumours included in the series were peripheral according to the above mentioned criteria (Mottura and Campobasso, 1966).

For the ultrastructural study, small samples from at least two different parts of each tumour were put in cold 6% glutaraldehyde in the operating theatre, immediately after surgery, and then cut into smaller fragments. These were fixed in 6% glutaraldehyde (Sabatini, Bensch and Barrnett, 1963), post-fixed in osmium tetroxide (Palade, 1952), and embedded in Araldite (Durcupan ACM Fluka) according to Luft (1961). Uranyl acetate was used during dehydration (Watson, 1958) and lead citrate applied to the sections (Reynolds, 1963) for staining. Semithin sections were stained with 1% toluidine blue, and specimens containing non-neoplastic tissue or suspected of it were discarded. Special care was also taken to avoid necrotic or fibrous areas. Thirteen tumours were found to be suitable for the electron microscopical investigation; thin sections obtained with the LKB Ultratome I or III were observed with the Elmiskope I Siemens electron microscope.

For light microscopy, several formalin-fixed paraffin-embedded blocks were made, some prepared from areas adjacent to those from which the specimens for electron microscopy had been chosen. Sections were stained with H. and E. and also with the method developed for lung tumours by Kreyberg and Jareg (Kreyberg, 1967). Tumours were classified according to the criteria of the W.H.O. (Kreyberg, 1967).

### RESULTS

### *Light microscopy*

The histological classification of the 13 peripheral lung tumours examined in the present series is recorded in Table I. According to the W.H.O. criteria, 5 tumours were classified as adenocarcinomata (Fig. 1, 2), 5 as large cell carcinomata (Fig. 3, 4), and 3 as epidermoid carcinomata. Among adenocarcinomata, one case only (Case 13) showed histological

TABLE I.—*Light and Electron Microscopic Correlations in Human Peripheral Lung Tumours*

| Case No. | Sex and Age | Light microscopy: W.H.O Classification | Keratin | Mucus | Electron microscopy group* |
|---|---|---|---|---|---|
| 1 | M 56 | Epidermoid carcinoma | ± | – | I |
| 2 | M 46 | Epidermoid carcinoma | ± | – | I |
| 3 | M 56 | Epidermoid carcinoma | ± | ± | I |
| 4 | M 45 | Large cell carcinoma giant-cell type | – | + | II |
| 5 | M 57 | Large cell carcinoma, solid type with mucin-like content | – | ++ | II |
| 6 | M 58 | Large cell carcinoma, solid type with mucin-like content | – | ++ | II |
| 7 | F 56 | Large cell carcinoma, solid type without mucin-like content | – | – | III |
| 8 | M 43 | Large cell carcinoma, solid type with mucin-like content | – | ++ | II |
| 9 | M 45 | Adenocarcinoma, bronchogenic acinar type | – | ++ | II |
| 10 | F 38 | Adenocarcinoma, bronchogenic acinar type | – | + | II |
| 11 | F 47 | Adenocarcinoma, bronchogenic papillary type | – | ++ | II |
| 12 | M 35 | Adenocarcinoma, bronchogenic papillary type | – | – | III |
| 13 | F 31 | Adenocarcinoma bronchiolo-alveolar type | – | – | III |

* See text.

FIG. 1.—Case 11. Adenocarcinoma, papillary type. H. and E. ×360.
FIG. 2.—Case 13. Adenocarcinoma, bronchiolo-alveolar type. H. and E. ×360.

FIG. 3.—Case 6. Large cell carcinoma, solid type with mucin-like content. H. and E. ×360.
FIG. 4.—Case 7. Large cell carcinoma, solid type without mucin-like content. H. and E. ×360.

FIG. 5.—Case 1 (epidermoid carcinoma): clear nucleus with sinuous outline, and cytoplasm with some bundles of tonofilaments (f); some nuclear sheets are present (large arrows). ×10,000.

FIG. 6.—Case 3 (epidermoid carcinoma): in the nucleus (N) a nucleolus with prominent nucleolonema (n); in the cytoplasm bundles of tonofilaments (f) and numerous ribosomes. ×17,000.

FIG. 7.—Case 3 (epidermoid carcinoma): a squamous cell with numerous tonofilament bundles (f); secretion vesicles (v). ×11,200.

FIG. 8.—Case 6 (mucus-secreting cell adenocarcinoma): a cavity between two cells bearing microvilli. ×14,000.

FIG. 9.—Case 11 (mucus-secreting cell adenocarcinoma): tumoural cells with many secretion vesicles (v), and microvilli. ×8000.

FIG. 10.—Case 9 (mucus-secreting cell adenocarcinoma): in the nucleus a nucleolus with prominent nucleolonema (n); secretion vesicles (v). ×14,400.

FIG. 11.—Case 9 (mucus-secreting cell adenocarcinoma). A cell bearing microvilli on the border of a glandular-like cavity, and showing some bundles of tonofilaments (f). ×8100.

Fig. 12.—Case 7 (alveolar cell adenocarcinoma): tumoural cells bearing microvilli, around a glandular-like cavity in which a lymphocyte and an erythrocyte are present; many osmiophilic lamellar (large arrows) and one non-lamellar (small arrow) bodies are present. ×5000.

Fig. 13.—Case 7 (alveolar cell adenocarcinoma): a tumoural cell bearing microvilli, around a glandular-like cavity; nucleus (N); an osmiophilic lamellar body (arrow) and numerous profiles of rough endoplasmic reticulum are evident. ×9,600.

Fig. 14.—Case 13 (alveolar cell adenocarcinoma): a tumoural cell with microvilli, abundant rough endoplasmic reticulum, and osmiophilic non-lamellar bodies. ×25,000.

patterns in keeping with the diagnosis of bronchiolo-alveolar carcinoma (Fig. 2). Among large cell carcinomata one tumour (Case 4) contained many very large or multinucleated cells and was classified as a giant cell type. In 3 out of 5 adenocarcinomata and in 4 out of 5 large cell carcinomata many mucus-secreting cells were present. A few mucus-secreting cells were scattered in the neoplastic tissues of one epidermoid carcinoma (Case 3). Keratinization was never seen in adenocarcinomata and large cell carcinoma, and it was scarce in epidermoid carcinomata.

*Electron microscopy*

Surgical material was found to be suitable for electron microscopy, even though tissue preservation was not always optimal. The most affected structures were often mitochondria which were probably damaged when some cells were rendered anoxic due to the clamping of blood vessels during surgery. Irrespective of their light microscopical classification, the 13 tumours were subdivided into 3 groups.

*Group I*.—In 3 tumours (Case 1, 2 and 3) the neoplastic tissue was characterized by a juxtaposition of large, irregular polygonal cells arranged in thick laminae and nests. The tumour cells had irregularly-shaped nuclei with sinuous outlines, and occasionally showed nuclear sheets (Fig. 5). The chromatin was finely dispersed, with a thin peripheral clumping (Fig. 5, 6); nucleoli with prominent nucleolonema could be seen (Fig. 6). The most characteristic cytological finding was the presence of prominent tonofilaments, often forming more or less thick bundles (Fig. 5, 6). Tonofilaments converged from the adjacent cytoplasm upon desmosomes which joined the cells together. Mitochondria were numerous, irregularly oval, and usually had a swollen appearance. Isolated or clumped ribosomes were numerous, and rough endoplasmic reticulum was variously developed. In one case (Case 3) some cells presented both tonofilaments and vesicles filled with rather translucent, amorphous looking material (Fig. 7).

*Group II*.—The cases included in this group (Case 4, 5, 6, 8, 9, 10 and 11) showed a glandular-like arrangement of the neoplastic tissue, due to the presence of more or less large cavities into which irregular microvilli passed from the neighbouring cells (Fig. 8, 9, 11). Desmosomes were present along the opposing plasma membranes, and tight junctions could be observed near to the free surfaces of the cells. The nuclei and nucleoli (Fig. 10, 11) were similar to those described for the cells of Group I. The cytoplasmic organization was characterized by the abundance of secretory vesicles in many cells (Fig. 9, 10). Tonofibrils often grouped in bundles were present in several cells (Fig. 11); secretory vesicles and tonofibrils could be observed occasionally in the same cell. Mitochondria were frequently swollen, and had irregular cristae. The Golgi apparatus was well developed, and the rough endoplasmic reticulum showed numerous profiles, often dilated and filled with granular material. In 2 cases (Case 6 and 11), a very few cells with osmiophilic lamellar bodies were occasionally found, similar to those which will be described in the next group.

*Group III*.—In the 3 cases (Case 7, 12 and 13) included in this group, neoplastic cells were arranged in glandular-like patterns, and joined by desmosomes and tight junctions. The nuclei (Fig. 12, 13) were round and darker than those of the cells of Groups I and II. The more striking and distinctive cytoplasmic feature of these cells was the presence of numerous peculiar dense bodies. These structures were mostly lamellar, with myelin-like figures often embedded in a homogeneous or vacuolated matrix (Fig. 12, 13). They were similar to the cytosomes of the type II normal alveolar cells (granular pneumocytes).

Smaller dense bodies with non-lamellar, though non-homogeneous, osmiophilic matrices (Fig. 12, 14) were also present and predominated in Case 13. Dense bodies showing intermediate patterns between lamellar and non-lamellar cytosomes were also observed. As in tumours of Group II, the cytoplasm was rich in free isolated and/or grouped ribosomes, and also in rough endoplasmic reticulum. Most of the mitochondria were swollen with myelin figures, and in this group of cases no cells containing secretory vesicles or tonofilaments were seen.

### DISCUSSION

The 13 peripheral lung tumours were classified under three independent headings after examination in light as well as by electron microscopy.

A good correlation between light and electron microscopy was observed for tumours classified as epidermoid carcinomata. At the ultrastructural level the cell arrangement, the richness in tonofilaments, and the nuclear patterns, were typical for squamous cells, and similar to the features usually reported for epidermoid non-keratinizing bronchogenic carcinomata (Greene, Brown and Divertie, 1969; Razzuk *et al.*, 1970).

On the other hand, both tumours diagnosed as adenocarcinoma and large cell carcinoma under light microscopy showed at the ultrastructural level a glandular-like pattern, due to the presence of cavities of variable size surrounded by epithelial cells whose free surfaces were provided with microvilli. The finding of microvilli demonstrates that these cavities are neither simple intercellular spaces, nor artificial effect of shrinkage: they must be regarded as true lumina of a glandular-like structure. The fact that this ultrastructural feature is common to adenocarcinoma and large cell carcinoma so designated at light microscopy level is much in keeping with the concept of other authors (Friedberg, 1965; Herman, Bullock and Waken, 1966) that large cell carcinomata, including giant cell cancer, are undifferentiated adenocarcinomata. This suggestion might well account for the difficulties in sharply separating adenocarcinoma and large cell carcinoma by light microscopy (Kreyberg, 1967; Campobasso, 1968; Melamed, 1968). It can be concluded that, irrespective of the light microscopical architecture, all peripheral lung tumours which fail to show squamous differentiation are in fact adenocarcinomata.

The distinction between Groups II and III was based mainly on the cytological characteristics. In Group II the prominent feature was the presence of cytoplasmic secretory vesicles, which may be related to the mucous secretion detected in all these tumours by light microscopy (Table I). In Group III the most characteristic cytological aspect was the presence of numerous osmiophilic cytosomes, with a lamellar or a dense pattern. Similar appearances of alveolar cell cytosomes have already been reported by many authors, and related partly to conditions of fixation, and partly to various developmental stages (Brooks, 1968; Flaks and Flaks, 1969). The occurrence of intermediate patterns between these two main types of dense bodies suggests that the non-lamellar bodies might be regarded as immature forms of alveolar lamellar cytosomes. This hypothesis is further supported by the findings of Johnston, Ginn and Amatulli (1971) in neoplastic elements from a metastatic alveolar cell carcinoma. A discussion on the development and significance of these lamellar bodies is beyond the aim of the present paper. They are, anyway, peculiar to granular pneumocytes (or type B or type II alveolar cells) and their presence in neoplastic cells has been regarded as ultrastructural evidence of the alveolar origin of both human and animal lung tumours (Klärner and Gieseking, 1960; Nagaishi *et al.*, 1965; Hattori *et al.*, 1967; Brooks, 1968; Adamson *et al.*, 1969; Flaks and Flaks, 1969; Johnston *et al.*, 1971; Nisbet *et al.*, 1971; Nash *et al.*, 1972). The tumours included in Group III of the present series must, therefore, be regarded as alveolar cell adenocarcinomata arising from granular pneumocytes.

These three tumours had been classified by light microscopy as: adenocarcinoma, bronchiolo-alveolar type (Case 13); adenocarcinoma, bronchogenic, papillary type (Case 12); and large cell carcinoma, without mucin-like content (Case 7). It must be concluded that the alveolar cell adenocarcinoma, if defined on the ultrastructural basis, may correspond to peripheral lung tumours of different histological type as defined by light microscopy. On the other hand, tumours included in Group II by electron microscopy had similar light microscope patterns to those of tumours included in Group III, nevertheless the former contained very occasional or no type II alveolar cells. It follows that electron microscopic examination is essential for the identification of the alveolar nature of lung tumours as no light microscopic pattern may *per se* reveal whether or not a lung tumour is made up of granular pneumocytes. This may well account for the conflicting ultrastructural findings in human tumours previously classified by light microscopy as alveolar cell cancer. In such tumours, cells bearing osmiophilic lamellar bodies were found by Adamson *et al.* (1969), Coalson *et al.* (1970), and Nash *et al.* (1972), but not by Geller and Toker (1969), and Razzuk *et al.* (1970).

However, it is noteworthy that the alveolar cell adenocarcinomata of the present series, besides being peripheral, shared the characteristic of being neither squamous nor mucus-secreting (Table I). A correlation between light and electron microscopy of peripheral lung adenocarcinomata seems, therefore, to exist as far as cytological characteristics are concerned. In fact, these tumours could be subdivided, on the basis of both light and electron microscopy, into mucus-secreting, and non-mucus-secreting adenocarcinomata, the latter corresponding to the tumours made up of malignant granular pneumocytes.

While the origin of the alveolar cell adenocarcinoma may be clearly identified by electron microscopy, the ultrastructural study has proved of limited value in elucidating the histogenesis of epidermoid and mucus-secreting peripheral lung tumours.

The cells of the mucus-secreting adenocarcinomata showed clear, irregularly-shaped nuclei, nucleoli with prominent nucleolonema, secretory vesicles and tonofilaments. All these cytological features were observed also in epidermoid carcinomata. Secretory vesicles were obviously much more prominent in the former, and tonofilament bundles in the latter. Nuclear sheets were observed only in epidermoid carcinomata, but this finding does not appear to be absolutely characteristic for any cell type (Mollo, Canese and Stramignoni, 1969).

The origin of both epidermoid and mucus-secreting peripheral lung tumours might be related to the epithelium of small bronchi as well as to the bronchiolar and/or alveolar epithelium; in fact the latter possesses a conspicuous proliferative capacity and " multipotential properties which include the production of mucous and stratified epithelium " (Spencer, 1968). An origin from the alveolar epithelium would appear more reasonable for mucus-secreting cell adenocarcinomata, as they share with alveolar cell adenocarcinomata several morphological aspects such as the presence of cavities surrounded by cells whose free surface is provided with microvilli, and also the occasional presence of cells containing osmiophilic lamellar bodies. However, as all the epithelial cells of the lower respiratory tract may undergo both squamous and mucous metaplasia, any hypothesis on the cell of origin of epidermoid and mucus-secreting peripheral tumours remains speculative.

The outcome of the present investigation is that human peripheral tumours can be classified, on the basis of both light and electron microscopy, as epidermoid carcinomata, mucus-secreting cell adenocarcinomata, and alveolar cell adenocarcinomata. Electron microscopy is required for identifying this third type of peripheral lung carcinoma, since it is not characterized by a single peculiar light microscopical appearance. However, the origin from granular pneumocytes may tentatively be suggested for peripheral lung tumours which appear neither squamous nor mucus-secreting in light microscopy.

### REFERENCES

ADAMSON, J. S., SENIOR, R. M. & MERRILL, T. (1969) Alveolar Cell Carcinoma. An Electron Microscopic Study. *Am. Rev. resp. Dis.*, **100**, 550.

BERKHEISER, S. W. (1966) Carcinoma *in situ* of the Lung of Peripheral (Bronchiolar) Origin. *Am. J. clin. Path.*, **46**, 315.

BROOKS, R. E. (1968) Pulmonary Adenomata of Strain A Mice: an Electron Microscopic Study. *J. natn. Cancer Inst.*, **41**, 719.

CAMPOBASSO, O. (1968) The Characteristics of Peripheral Lung Tumours that Suggest their Bronchiolo-alveolar Origin. *Br. J. Cancer*, **22**, 655.

COALSON, J. J., MOHR, J. A., PIRTLE, J. K., DEE, A. L. & RHOADES, E. R. (1970) Electron Microscopy of Neoplasms in the Lung with Special Emphasis on the Alveolar Cell Carcinoma. *Am. Rev. resp. Dis.*, **101**, 181.

FLAKS, B. & FLAKS, A. (1969) Fine Structure of Murine Pulmonary Adenomata Induced by Carcinogen Treatment in Organ Culture. *Cancer Res.*, **29**, 1781.

FRIEDBERG, E. C. (1965) Giant Cell Carcinoma of the Lung. A Dedifferentiated Adenocarcinoma. *Cancer, N.Y.*, **18**, 259.

GELLER, S. A. & TOKER, C. (1969) Pulmonary Adenomatosis and Peripheral Adenocarcinoma of the Lung. An Ultrastructural Demonstration of Common Morphologic Features. *Archs Path.*, **88**, 148.

GREENE, J. G., BROWN, A. L. & DIVERTIE, M. B. (1969) Fine Structure of Squamous Cell Carcinoma of the Lung. *Proc. Staff Meet. Mayo Clin.*, **44**, 85.

HATTORI, S., MATSUDA, M., TATEISHI, R. & TERAZAWA, T. (1967) Electron Microscopic Studies on Human Lung Cancer Cells. *Gann*, **58**, 283.

HERMAN, D. L., BULLOCK, W. K. & WAKEN, J. K. (1966) Giant Cell Adenocarcinoma of the Lung. *Cancer, N.Y.*, **19**, 1337.

JOHNSTON, W. W., GINN, F. L. & AMATULLI, J. M. (1971) Light and Electron Microscopic Observations on Malignant Cells in Cerebrospinal Fluid from Metastatic Alveolar Cell Carcinoma. *Acta cytol.*, **15**, 365.

KLÄRNER, P. & GIESEKING, R. (1960) Zur Ultrastruktur des Lungentumors der Maus. *Z. Krebsforsch.*, **64**, 7.

KREYBERG, L. (1967) *Histopathological Typing of Lung Tumours.* Geneva: W.H.O.

LISA, J. R., TRINIDAD, S. & ROSENBLATT, M. B. (1965) Site of Origin, Histogenesis and Cytostructure of Bronchogenic Carcinoma. *Am. J. clin. Path.*, **44**, 375.

LUFT, J. H. (1961) Improvements in Epoxy Resin Embedding Methods. *J. biophys. biochem. Cytol.*, **9**, 409.

MELAMED, M. R. (1968) Pathology. In *Lung Cancer. A Study of Five Thousand Memorial Hospital Cases.* Ed. W. L. Watson. St Louis: C. V. Mosby.

MEYER, E. C. & LIEBOW, A. A. (1965) Relationship of Interstitial Pneumonia Honey-combing and Atypical Epithelial Proliferation to Cancer of the Lung. *Cancer, N.Y.*, **18**, 322.

MOLLO, F., CAMPOBASSO, O. & CANESE, M. G. (1967) Ricerche di microscopia elettronica sulle cellule di tumori polmonari periferici in riferimento al problema istogenetico. In *Atti X Congr. Soc. Ital. Patol.*, IDOS, Milano. Part II, p. 869.

MOLLO, F., CANESE, M. G. & STRAMIGNONI, A. (1969) Nuclear Sheets in Epithelial and Connective Tissue Cells. *Nature, Lond.*, **221**, 869.

MOTTURA, G. & CAMPOBASSO, O. (1966) Sui criteri morfologici e istogenetici per la classificazione dei carcinomi broncopolmonari. *Minerva pneumol.*, **5**, 40.

NAGAISHI, C., OKADA, Y., DAIDO, S., GENKA, K., IKEDA, S. & KITANO, M. (1965) Electron Microscopic Observations of the Human Lung Cancer. *Expl. Med. Surg.*, **23**, 177.

NASH, G., LANGLINAIS, P. C. & GREENAWALD, K. A. (1972) Alveolar Cell Carcinoma: Does it Exist? *Cancer, N.Y.*, **29**, 322.

NISBET, D. I., MACKAY, J. M. K., SMITH, W. & GRAY, E. W. (1971) Ultrastructure of Sheep Pulmonary Adenomatosis (Jaagsiekte). *J. Path. Bact.*, **103**, 157.

PALADE, G. E. (1952) A Study of Fixation for Electron Microscopy. *J. exp. Med.*, **95**, 285.

RAZZUK, H. A., RACE, G. J., LYNN, J. A., MARTIN, J. A., URSCHEL, H. C. & PAULSON, D. L. (1970) Observations on Ultrastructural Morphology of Bronchogenic Carcinoma. *J. thorac. cardiovasc. Surg.*, **59**, 581.

REYNOLDS, E. S. (1963) The Use of Lead Citrate at High pH as an Electron-opaque Stain in Electron Microscopy. *J. Cell Biol.*, **17**, 208.

SABATINI, D. D., BENSCH, K. & BARRNETT, R. J. (1963) Cytochemistry and Electron Microscopy. The Preservation of Cellular Structure and Enzymatic Activity by Aldehyde Fixation. *J. Cell. Biol.*, **17**, 19.

SASAKI, M., HAYASHI, N. & YAMORI, T. (1964) Electron Microscopic Studies on Human Pulmonary Carcinoma. *Gann*, **55**, 109.

SCHULZ, H. (1963) Some New Observations on the Submicroscopic Pathology of the Lung; Pulmonary Adenomatosis and Fat Embolism. *Lab. Invest.*, **12**, 616.

SPENCER, H. (1968) *Pathology of the Lung*, 2nd ed. Oxford: Pergamon.

WATSON, M. L. (1958) Staining of Tissue Sections for Electron Microscopy. *J. biophys. biochem. Cytol.*, **4**, 475.